# Sensing performances of pure and hybridized carbon nanotubes-ZnO nanowire networks: A detailed study

**DOI:** 10.1038/s41598-017-14544-0

**Published:** 2017-11-07

**Authors:** Oleg Lupan, Fabian Schütt, Vasile Postica, Daria Smazna, Yogendra Kumar Mishra, Rainer Adelung

**Affiliations:** 1 Institute for Materials Science, Christian-Albrechts Universität zu Kiel, Kaiser Str. 2, D-24143 Kiel, Germany; 2 Department of Microelectronics and Biomedical Engineering, Technical University of Moldova, 168 Stefan cel Mare Av., MD-2004 Chisinau, Republic of Moldova

## Abstract

In this work, the influence of carbon nanotube (CNT) hybridization on ultraviolet (UV) and gas sensing properties of individual and networked ZnO nanowires (NWs) is investigated in detail. The CNT concentration was varied to achieve optimal conditions for the hybrid with improved sensing properties. In case of CNT decorated ZnO nanonetworks, the influence of relative humidity (RH) and applied bias voltage on the UV sensing properties was thoroughly studied. By rising the CNT content to about 2.0 wt% (with respect to the entire ZnO network) the UV sensing response is considerably increased from 150 to 7300 (about 50 times). With respect to gas sensing, the ZnO-CNT networks demonstrate an excellent selectivity as well as a high gas response to NH_3_ vapor. A response of 430 to 50 ppm at room temperature was obtained, with an estimated detection limit of about 0.4 ppm. Based on those results, several devices consisting of individual ZnO NWs covered with CNTs were fabricated using a FIB/SEM system. The highest sensing performance was obtained for the finest NW with diameter (D) of 100 nm,  with a response of about 4 to 10 ppm NH_3_ vapor at room temperature.

## Introduction

Functionalization of semiconducting oxides via hybridizing them with appropriate metallic or carbon nanostructures is known to be a very powerful strategy to strongly enhance and likewise to control the oxides’ electrical, chemical and physical properties, particularly nanoscopic dimensions and hence adequate applications^1–6^. Thus, these approaches are widely adopted and investigated, especially in the field of new nanomaterials for sensing applications^2,3,5,7–10^. The combination of two materials often leads to apparition of new and unique effects. Due to excellent sensing properties of carbon based nanomaterials (e.g. CNTs) in detection of NO_2_ and NH_3_ at room temperature, the combination of CNTs and metal oxides is widely used for fabrication of highly selective sensors operating at room temperatures. Additionally, monitoring of NH_3_ gas for indoor and outdoor applications is a very important task due to an increasing rate of atmospheric pollution with ammonia in recent years^11^. For example, Wei *et al*. observed that hybrid CNT/SnO_2_ gas sensors exhibit much higher sensitivity and recovery properties in detecting of NO_2_ gas at room temperature than a pristine SnO_2_ sensor^12^. Deng *et al*. fabricated reduced graphene oxide (rGO) conjugated Cu_2_O NW mesocrystals for high-performance NO_2_ gas sensors^13^. Van Hieu *et al*. demonstrated much better response and sensing rapidity to NH_3_ gas of SnO_2_/multiwalled carbon nanotubes (MWCNT) composites compared to pristine SnO_2_ or CNT structures^14^. Therefore, these carbon-nanotubes based hybrid nanomaterials are going to play a lead role towards reliable sensing devices.

Another important advantage of hybridization of semiconducting oxides and CNT heterostructures is their high efficiency in separation of photogenerated electron-hole pairs, for example in ZnO^15^. Dutta and Basak reported the fabrication of MWCNTs/ZnO NW composites with enhanced UV sensing properties which were attributed to surface plasmon resonance mediating a rapid electron transfer between ZnO and MWCNTs^7^.  Furthermore, Wang *et al*. fabricated high-performance UV sensors based on a rGO decorated hydrangea-like ZnO film on a PDMS substrate for flexible electronics^16^. Another interesting UV sensing application was demonstrated by Jin *et al*. by fabrication of high-performance UV photodetectors based on graphdiyne: ZnO nanocomposites^2^. Nie *et al*. fabricated high-performance Schottky junction UV photodetectors based on a monolayer graphene film on a ZnO nanorod array^3^.

However, in the field of sensing applications, very little attention has been paid to single hybrid nanostructures based devices. Individual nanostructures are known for their advantageous high surface-to-volume ratio^17,18^. Thus, the charge transport through the nanostructures is very sensitive to surface phenomena, making them ideal candidates for gaseous and biological sensing applications^19,20^. Some representative examples of integration of hybrid nanostructures in nanosensors were recently reported. For example, fabrication of a hybrid nanosensor based on the electrochemical reduction of TNT and the interaction of the reduction products with conducting polymer nanojunctions in an ionic liquid was reported^21^. Lupan *et al*. integrated single ZnO tetrapods and microwires (MW) functionalized with Fe_2_O_3_ nanoparticles into a nanosensor for enhanced ethanol sensing properties^10^. The devices based on single Bi_2_O_3_/ZnO MW showed an enhanced H_2_ gas response compared to pristine ZnO MWs^1^. Furthermore, functionalized MWCNTs with Ag nanocrystals have been displaying a considerable enhancement in NH_3_ response^22^.

However, to the best of our literature knowledge, no reports on gas sensing properties of individual carbon based nanomaterials hybridized metal oxide nanostructures were found. In this work, highly porous (~93%) ZnO NW networks were hybridized with CNTs. For the first time individual ZnO-CNT NWs with different diameters (down to 100 nm) were integrated into nanosensor devices in order to develop a highly sensitive nanosensor with high selectivity to NH_3_. The UV and gas sensing properties of individual and networked ZnO-CNT NWs were investigated by varying the content of CNTs from 0.4 to 4.0 wt%, showing an improvement in sensing properties by increasing the content of CNTs, having an optimum at 2.0 wt%. Furthermore, the influence of relative humidity on sensing properties was studied in detail. UV and gas sensing mechanisms based on energy band diagrams were proposed and discussed in detail for individual and networked hybrid NWs.

## Results

### Morphologies of CNT functionalized ZnO nanowire networks

**Figure 1:**
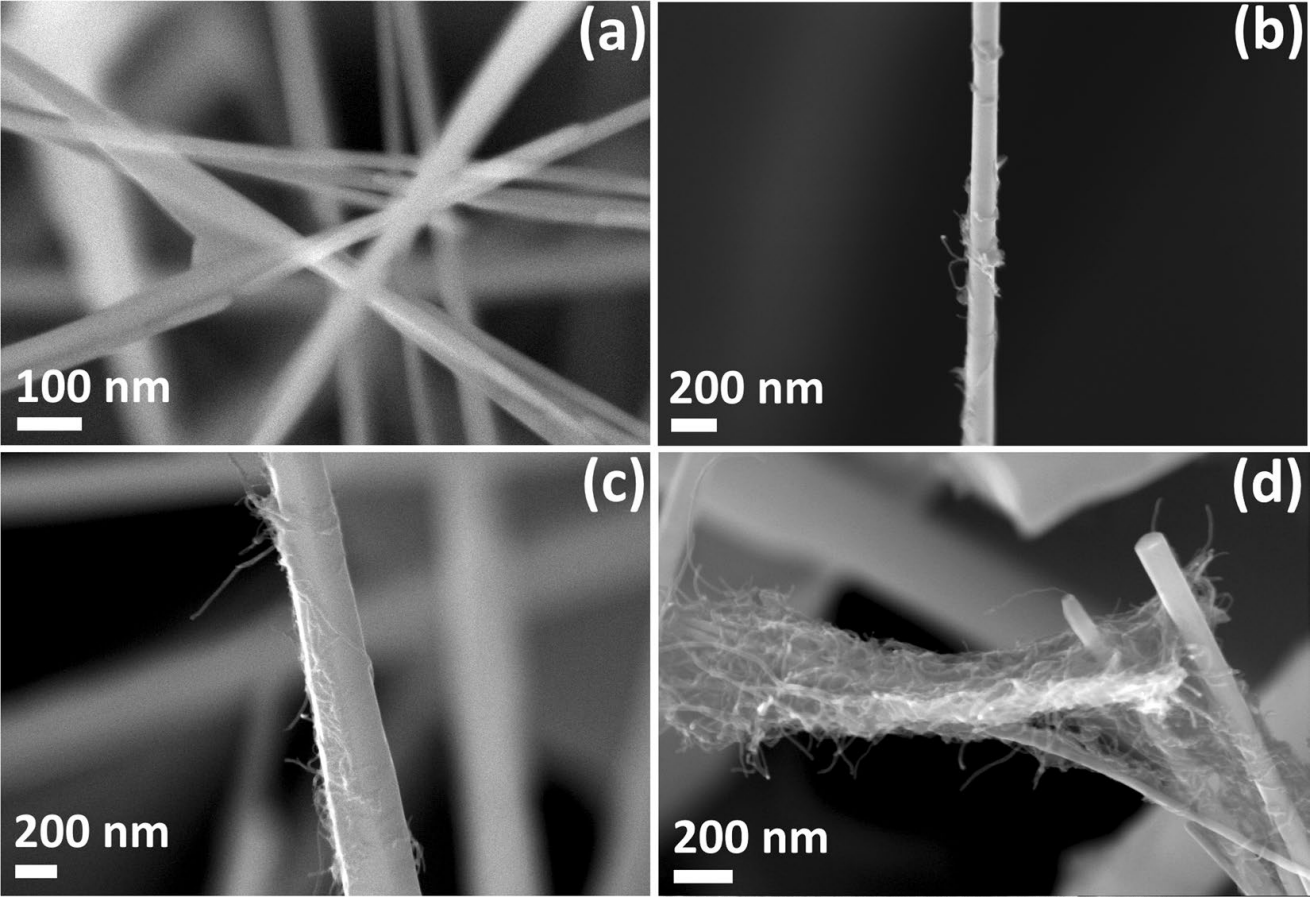
Morphological evolutions of pure and CNT hybridzed ZnO nanowire networks: SEM images of ZnO-CNT NWs with: (**a**) 0.0 wt% CNT; (**b**) 0.4 wt% CNT; (**c**) 2.0 wt% CNT; and (**d**) 4.0 wt% CNT.

Figure 1 shows the high magnification SEM images of ZnO-CNT NWs synthesized in this work with different content of CNTs (from 0.0 to 4.0 wt% CNT) in order to demonstrate that CNTs were successfully attached to the surface of ZnO NWs. The details on synthesis method and detailed characterization of ZnO NW networks, as well as the influence of synthesis parameters on morphology of the networks were presented in previous works^23–25^ (Fabian Schütt, Stefano Signetti, Helge Krüger, Sarah Röder, Daria Smazna, Sören Kaps, Stanislav N. Gorb, Yogendra Kumar Mishra, Nicola M. Pugno, Rainer Adelung Nat. Commun. 2017 in press, doi:10.1038/s41467-017-01324-7). In the present work, we used ZnO NW networks synthesized in a N_2_ atmosphere at 900 °C. The diameter of the as fabricated NWs is in the range of 50–300 nm while their length is up to several tens of micrometers (see Fig. 1). The produced NW powder was further pressed into a cylindrical shape, which was sintered at 1150 °C for 5 h to produce an interconnected 3D network with a density of 0.3 g/cm³. Due to the hydrophilic character and the high porosity (93%), these networks can be easily infiltrated with an aqueous CNT dispersion (0.1 wt%) using a simple dripping procedure. The diameter of the used CNTs is in range of 10–30 nm, while their length is up to several microns (Supplementary Fig. S1a). Upon drying, the CNTs form a homogenous layer around the interconnected NWs (Fabian Schütt, Stefano Signetti, Helge Krüger, Sarah Röder, Daria Smazna, Sören Kaps, Stanislav N. Gorb, Yogendra Kumar Mishra, Nicola M. Pugno, Rainer Adelung Nat. Commun. 2017 in press, doi:10.1038/s41467-017-01324-7). Next to the homogenous layer, also some CNT nets are formed between the NWs during drying. By repeated drying and infiltration the amount of CNTs can be varied. After hybridization with CNTs, no change in the morphology of the ZnO NWs was observed. As can be seen in Fig. 1, an increase in infiltration times leads to a gradual increase of CNTs on the surface of ZnO NWs (from 0.4 to 4.0 wt%). In the case of networks with 4.0 wt% CNT percolating networks of CNTs are formed between the NWs (see Fig. 1d). Thus, it can be concluded that the described synthesis strategy leads to an excellent surface functionalization of ZnO NW with CNTs (see Fig. 1b–d). From Fig. 1c, it can be clearly observed that CNTs surround the entire ZnO NWs with good adhesion, which is rather very important for further integration of individual NWs into nanosensor devices^26^. Thus, only a negligible amount of CNTs will detach, which will be further demonstrated. Due to the homogenous coating, the calculated CNT concentration with respect to the ceramic network can be taken as an estimated value for the amount of CNTs on a single NW. More SEM images of ZnO-CNT NWs with 2.0 wt% CNT are shown in Supplementary Fig. S1b–d.

### UV sensing properties of hybrid ZnO NW networks

**Figure 2:**
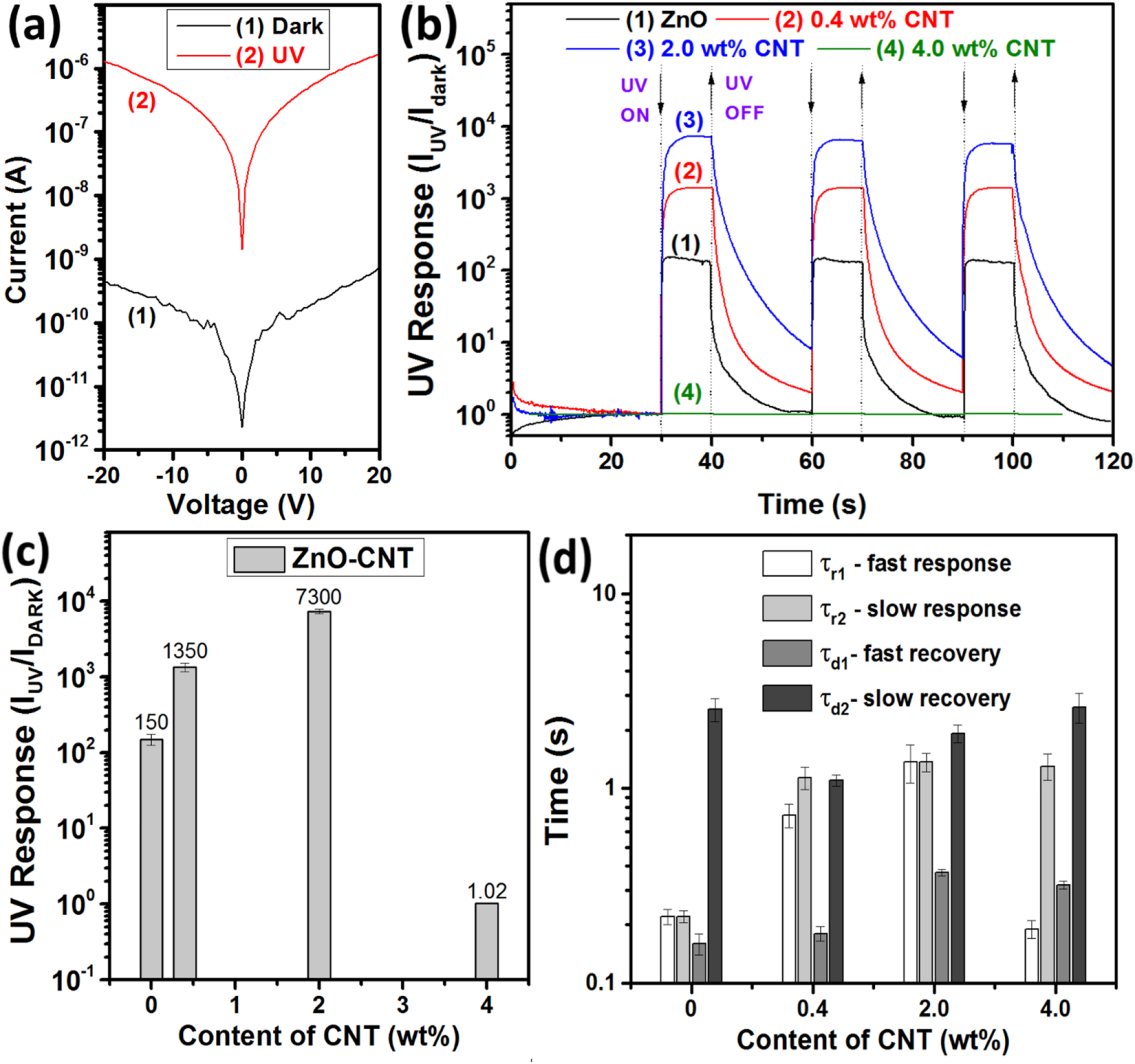
(**a**) Current – voltage characteristics of the device based on ZnO-CNT networks with 2.0 wt% CNT. (**b**) Dynamic UV response of ZnO-CNT networks with different content of CNT (C in wt%) at 10 V applied bias voltage. (**c**) UV response versus content of CNT in ZnO-CNT networks (C in wt%). (**d**) Calculated rise (*τ*_r1_ and *τ*_r2_) and decay (*τ*_d1_ and *τ*_d2_) time constants from UV response curves using bi-exponential fitting.

Figure 2a shows the current – voltage (*I-V*) characteristics of a device fabricated using ZnO-CNT networks with 2.0 wt%CNT, showing double Schottky contacts formation and a high UV on/off current ratio. Figs 2b and S1e show the dynamic UV response of devices fabricated using pristine ZnO NW networks and networks which were functionalized with CNTs to three pulses of UV light with 10 s durations and 30 s periods, respectively. The bias voltage applied on to the device structure was 10 V. The addition of CNTs to ZnO NW networks (up to 2.0 wt%) resulted in an increase of dark current (not shown) which can be attributed to the excellent electrical conductivity of CNTs. By adding 4.0 wt% CNT, the resistance of networks decreased dramatically leading to a higher dark current and poor UV detection properties (shown in Fig. 2b). This can be accounted for the formation of the percolating carbon nanotube networks which considerably reduce the influence of potential barriers between ZnO NWs on conductivity, thereby lowering the adsorption sites for oxygen molecules^7^. However, the high rapidity of the networks was not affected. The calculated UV response for each type of samples is presented in Fig. 2c. The pristine ZnO NW networks demonstrated a low UV response of about 150. By adding 2.0 wt% of CNTs the UV response was considerably increased to 7300 (about 50 times). This value is much higher than any reported studies ever for MWCNT/ZnO NWs^7^ and rGO decorated ZnO nanostructures^16^. The UV response of samples with high content of CNTs (4.0 wt%) showed very low UV response of about 1.02. In order to evaluate the rapidity of samples, the rise and decay time components were calculated by bi-exponential fitting (equations (1 and 2))^27,28^:

1 $$I(t)={I}_{dark}+{A}_{1}(1\,-\,{e}^{-\frac{t}{{\tau }_{r1}}})+{A}_{2}(1\,-\,{e}^{-\frac{t}{{\tau }_{r2}}})$$

2 $$I(t)={I}_{dark}+{A}_{3}{e}^{-\frac{t}{{\tau }_{d1}}}+{A}_{4}{e}^{-\frac{t}{{\tau }_{d2}}}$$

where *A*_1_, *A*_2_, *A*_3_ and *A*_4_ are positive constants, *τ*_r1_ and *τ*_r2_ are fast and slow time constants for rising photocurrent, *τ*_d1_ and *τ*_d2_ are fast and slow time constants for decaying photocurrent, respectively. The calculated results are presented in Fig. 2d, while the examples of bi-exponential fitting of response curves are presented in Supplementary Fig. S2. As can be observed, the *τ*_d2_ time constant of UV response is significantly improved by adding CNT up to 0.4 wt%. For the pristine ZnO NW networks the *τ*_r1_, *τ*_r2_, *τ*_d1_ and *τ*_d2_ values are 0.22, 0.22, 0.16 and 2.55 s, respectively, while for samples with 2.0 wt% the *τ*_r1_, *τ*_r2_, *τ*_d1_ and *τ*_d2_ is 1.37, 1.37, 0.37 and 1.92 s, respectively. The increase in rise time for samples with 0.4 and 2.0 wt% CNT can be referred to decreased porosity and lowering of the energy of adsorption sites for oxygen molecules^7,16^. However, the rapidity of our networks is superior to the rapidity of MWCNTs coated ZnO NW arrays^7^.

The responsivity (*R*) and internal photoconductive gain (*G*) were calculated using the following equation (3)^10,29,30^:

3 $$R=\frac{{I}_{ph}}{{P}_{opt}}=\eta (\frac{q\lambda }{hc})G$$

where *I*_ph_ is the photocurrent (*I*_UV_ − *I*_dark_), *P*_opt_ is incident optical power of the UV source, *η* is quantum efficiency (for simplicity was set to 1), *h* is Planck’s constant, *c* is the speed of light, *λ* is wavelength of UV light (365 nm). The calculated values of *R* for pristine ZnO NW networks and ZnO-CNT networks with 0.4, 2.0 and 4.0 wt% CNT are 1.5 × 10^−5^, 8.6 × 10^−5^, 2.4 × 10^−4^, 4.8 × 10^−4^ A·W^−1^, respectively, while the calculated *G* values are 5.1 × 10^−5^, 2.9 × 10^−4^, 8.1 × 10^−4^, 1.6 × 10^−3^, respectively. Such low values of responsivity and internal photoconductive gain can be associated with relatively high UV light intensity and area of sensing material (~0.01 cm^2^). However, the increase in *R* and *G* by rise in CNTs content can be due to the prolonged photocarrier lifetime (*τ*). It is known that the internal photoconductive gain mainly depends on the *τ*, as represented by equation (4)^10,29,30^:

4 $$G\cong \frac{1}{{L}^{2}}\tau {\mu }_{e}V$$

where *L* is the interelectrode spacing, *μ*_*e*_ is the electron mobility and *V* is the applied bias voltage. Thus, the presence of CNTs on the surface of ZnO NWs prolongs the photocarrier lifetime due to an easy transfer of the photogenerated electrons to the CNTs and prevents electron-hole recombination (see Supplementary Fig. S3)^15,30,31^. Vietmeyer *et al*. determined that the average rate constant for electron transfer between excited ZnO and CNT is 1 × 10^8^ s^−1^ ^31^. Thus, more photogenerated holes can migrate to the surface of ZnO NWs and discharge the adsorbed oxygen molecules by surface electron-hole recombination^30,32^. Mu *et al*. also observed for ZnO-carbon nanofibers hybrid structures a great inhibition of recombination processes of the photogenerated charge carriers^15^. The increased UV response can be also explained based on the excellent ZnO-CNT hybrid structures separation properties of photogenerated electron-hole pairs (see Supplementary Fig. S3)^16,31^. The influence of applied bias voltage and RH is discussed in Supplementary Text S3.

### Gas sensing response of hybrid CNT-ZnO NW networks

**Figure 3:**
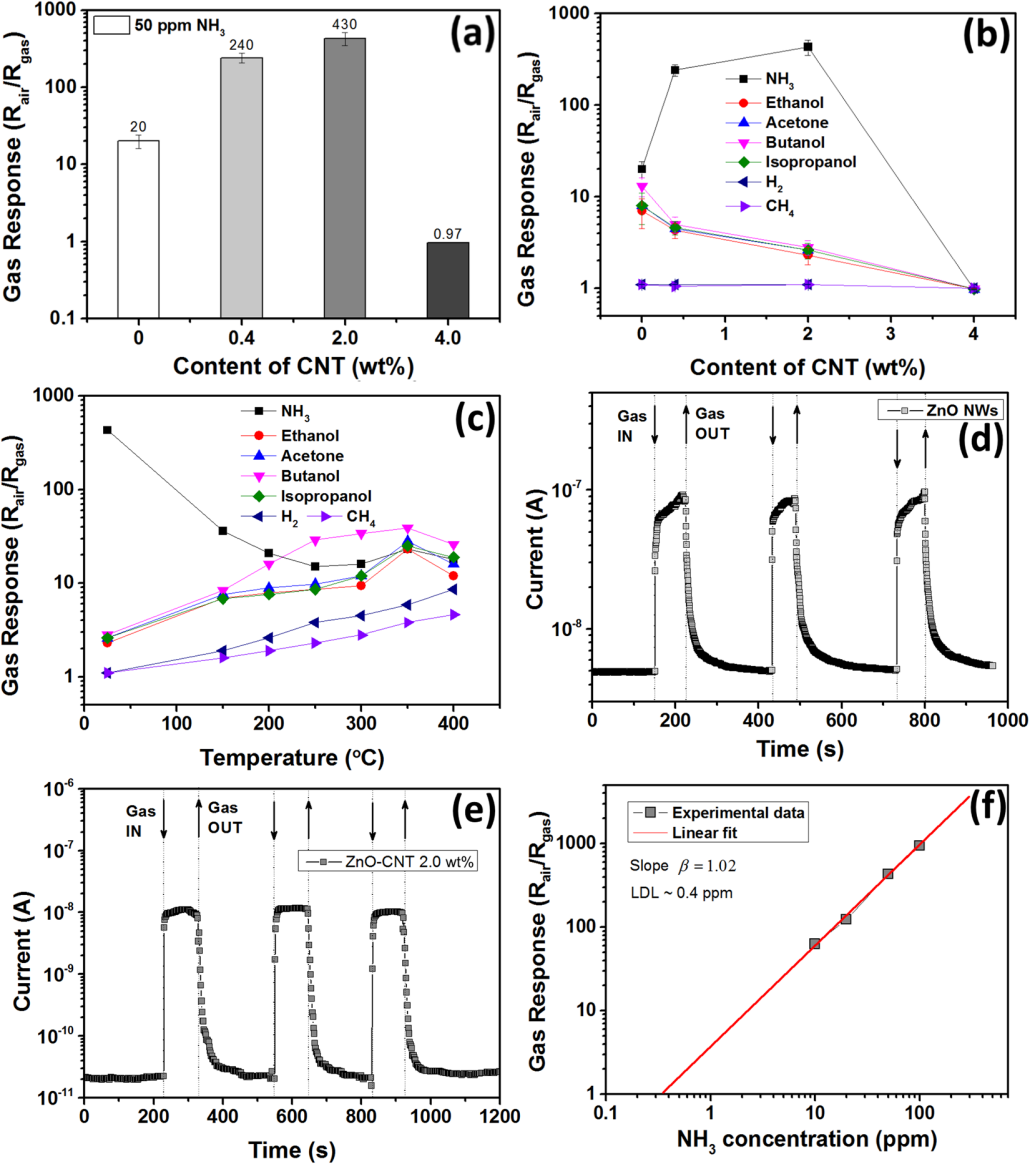
(**a**) The gas response of devices with different content of CNT to 50 ppm of NH_3_ at room temperature. The gas response of devices with different content of CNT at room temperature (**b**); and the gas response of sample with 2.0 wt% CNT versus operating temperature (**c**) to 50 ppm of NH_3_, 100 ppm of ethanol, acetone, butanol and isopropanol and 10 000 ppm of H_2_ and CH_4_. Dynamic gas response of devices with different content of CNT at room temperature to 50 ppm of NH_3_: (**d**) ZnO; and (**e**) ZnO-CNT with 2.0 wt%. (**f**) Gas response at room temperature versus concentration of NH_3_ for sample with 2.0 wt% CNT.

The addition of carbon nanomaterial to metal oxide nanostructures is known to be a powerful tool to enhance ammonia response at room temperature^12^. Thus, we explore for the first time new developed nano-devices for gas sensing applications. Figure 3a shows the room temperature gas response to 50 ppm of NH_3_ for devices fabricated using samples with different content of CNTs. The gas response is considerably increasing from 20 to 240 and 430 by increasing the CNT amount from 0 to 0.4 wt% and 2.0 wt%, respectively. Thus, an increase in response by more than 20 times was obtained by hybridizing with 2.0 wt% CNTs in ZnO NW networks. In the case of samples with 4.0 wt% CNTs the typical *p*-type response of carbon based nanomaterials was observed (see Supplementary Fig. S5a)^33^. This effect is due to high concentration of CNTs which form percolating networks and dominate the gas response^14^. Therefore, due to the poor gas sensing performance of these samples, they will be excluded from further investigations. Beside the high NH_3_ response at room temperature, an excellent selectivity of ZnO-CNT was also observed. Figure 3b shows the gas response to other tested gases (H_2_ and CH_4_ with concentration of 10 000 ppm) and volatile organic compounds (VOCs, ethanol, acetone, butanol and isopropanol with concentrations of 100 ppm) versus content of CNT . For pristine ZnO NW networks a low selectivity to NH_3_ was observed. The gas response to ethanol, acetone, butanol, isopropanol, H_2_ and CH_4_ is 7, 8, 13, 8, 1.1 and 1.1, respectively. By using CNT – hybridization of ZnO NW networks the decrease in gas response to these gases and VOCs can be observed. No response higher than 3 was measured for ZnO-CNT networks with 2.0 wt% at room temperature, demonstrating the improved selectivity of the 3D networks to NH_3_. By increasing the operating temperature up to 400 °C, a considerable decrease in gas response to NH_3_ and selectivity was observed due to an increase in gas response to other tested gases and VOCs (see Fig. 3c). The gas response to all gases and VOCs at high operating temperatures can be explained based on a conventional ionosorption mechanism, i.e., oxidation of gas and VOCs molecules by adsorbed oxygen species at the surface of ZnO^34^, which was described in detail in our previous works for different morphologies of ZnO micro- and nanostructures as well as single structures^25,27,32^, while low selectivity of pure ZnO is a well known fact^35^.

The decrease in response to VOCs by adding CNTs can be clarified taking into account lowering of the number of adsorption sites for oxygen species, therefore lowering the overall gas response^7,14^. For example, Lupan *et al*. observed that by adding an excessive content of CuO nanomaterial to ZnO tetrapod networks leads to a lowering of its porosity and thus a decrease in gas response^6^. The same holds for an excessive addition of Bi_2_O_3_^1^. Yu *et al*. also observed that excessive addition of Fe_2_O_3_ to ZnO nanostructures leads to reduction in coverage of oxygen species^36^. However, in our case the NH_3_ response is increasing by adding CNT up to 2.0 wt% (see Fig. 3a). This can be explained on the basis of excellent sensing properties of CNTs and other carbon based nanomaterials to selectively detect NH_3_ molecules^37,38^. Bardley *et al*. calculated that adsorption of one NH_3_ molecule leads to a 0.04 e^−^ transfer to CNT^39^. Due to lower work function of CNT compared to those of ZnO^40,41^, the electrons can easily travel between materials and change the concentration of charge carriers in ZnO NWs. For example, Lu *et al*. fabricated a room-temperature gas sensor based on discrete SnO_2_ nanocrystals and MWCNTs and explained the improved gas sensing properties based on electron transfer between the materials^42^. Also, the high selectivity to ammonia at low temperatures can be explained based on the low ionization energy (10.18 eV) and kinetic diameter (0.36 nm) of ammonia compared to other vapors, as well as the higher electron donating ability of ammonia^43^.

**Figure 4:**
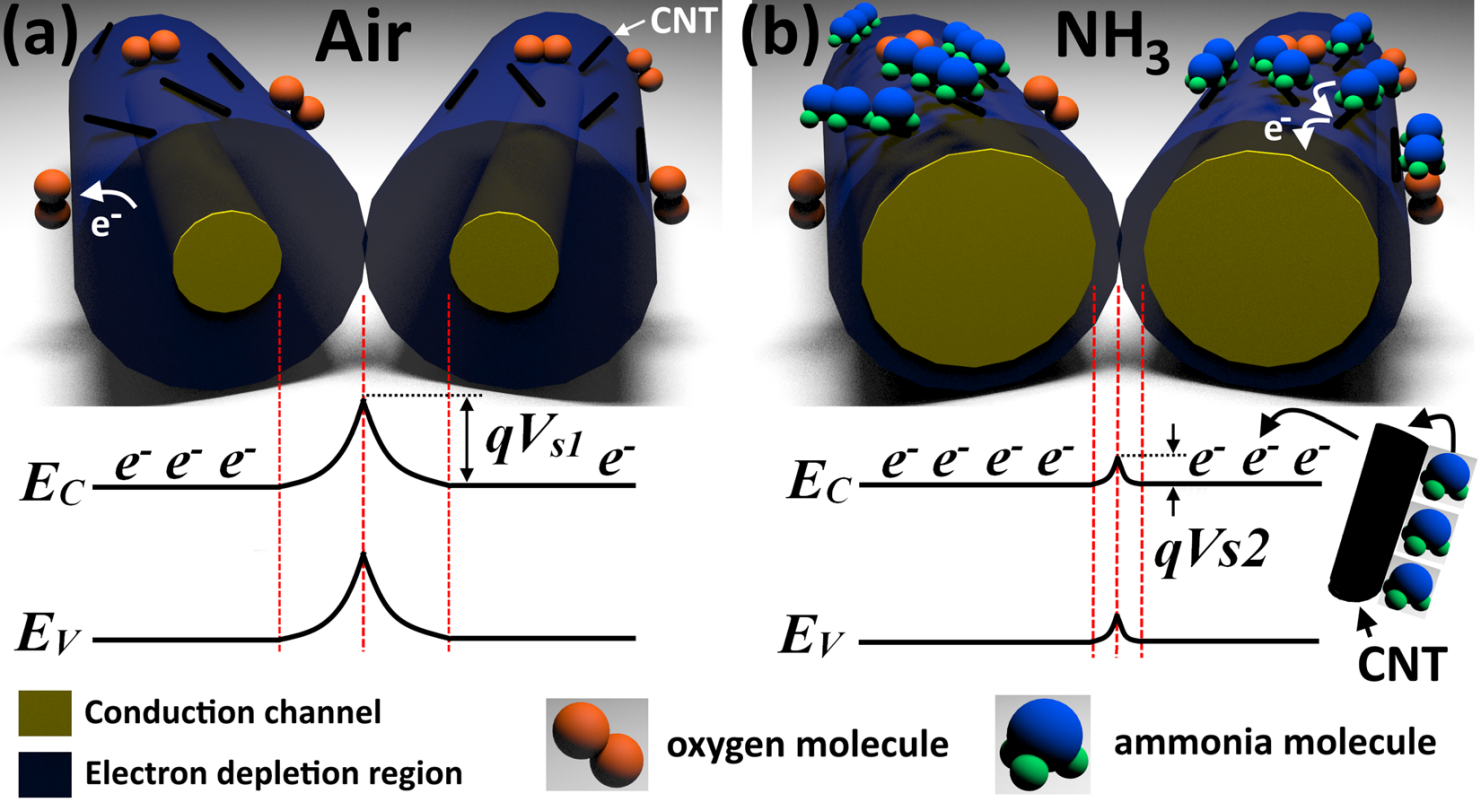
Proposed gas sensing mechanism with energy band diagrams to NH_3_ at room temperature for ZnO-CNT networks without taking in account the oxidation of NH_3_ molecules by oxygen species. (**a**) At exposure to air the oxygen molecules are adsorbed on the surface of ZnO NWs forming an electron depletion region (by ionization) and pottential barrier at the interface of NWs with height *qV*_s1_. (**b**) Under exposure to ammonia, the NH_3_ molecules are adsorbed on the CNT surface and transfer the donated electrons to the ZnO NWs. This leads to a narrowing of the electron depletion region and lowering of the potential barrier height *qV*_s2_.

In our case under exposure to NH_3_, the ammonia molecules are adsorbed on the surface of the CNT leading to a subsequent transfer of electrons to the underlying ZnO NWs, thereby increasing the number of charge carriers *N*_*D*_ (see Fig. 3d). Due to the potential barriers formed between ZnO NWs under exposure to air (*qV*_s1_) being sensitive to *N*_*D*_ (see Fig. 4a and equation (5))^44^, a decrease in height of *qV*_s1_ to *qV*_s2_ under exposure to NH_3_ can be obtained, causing an increase of current through the networks (see Figs 3d and 4b). The dependence of gas response on the change of the potential barriers height is given by equations (5 and 6)^45^:

5 $${V}_{s1}=\frac{2\pi {Q}_{s}^{2}}{\varepsilon {N}_{D}}$$

6 $$S=\frac{{R}_{air}}{{R}_{gas}}\approx exp(\frac{q({V}_{s1}\,-\,{V}_{s2})}{kT})$$

where *Q*_*s*_ is the surface charge density, *T* is the operating temperature, *q* is the elementary charge, *k* is the Boltzmann constant and *ε* is the dielectric constant of ZnO. Thus, by adding more CNTs to the highly porous ZnO NW networks, a higher electron transfer upon exposure to NH_3_ can be achieved, causing a higher gas response . In general, such high gas response is related to the high porosity of the ceramic networks, which facilitate the diffusion of gaseous species and the thin diameter of ZnO NWs (in range of 50–300 nm, see Fig. 1)^26^.

Next, the dynamic properties of ZnO-CNT networks will be discussed. Figure 3d shows the dynamic gas response to 50 ppm of NH_3_ at room temperature of devices fabricated using pristine ZnO NWs, while Fig. 3e shows the dynamic response of a device fabricated using networks with 2.0 wt% CNT. The calculated response time and recovery time (defined as time to obtain and recover 90% of the signal, respectively) are 53 s and 34 s for pristine ZnO networks, respectively, and 25 s and 18 s for samples with 2.0 wt% CNT, respectively. The calculated data for all samples are presented in Supplementary Fig. S5b, showing the increase in rapidity by rise in content of CNTs up to 2.0 wt%, while for samples with 4.0 wt% CNT the recovery time is much higher, comparable with those of carbon based nanomaterial sensors^37,38^. Figure 3f shows the gas response of samples with 2.0 wt% CNT versus the concentration of NH_3_, showing a typical power law dependence^46^. The calculated slope is *β* = 1.02 and the lowest detection limit (LDL) is about 0.4 ppm (taking into account that criterion for gas detection is *R*_air_ */R*_gas_ > 1.2)^47^. The gas responses to 10, 20, 50, and 100 ppm are 63, 124, 430 and 940, respectively. The influence of high RH values on gas response was also investigated, see Supplementary Fig. S5c,d.

### Single hybrid CNT-ZnO nanowire based sensor device

**Figure 5:**
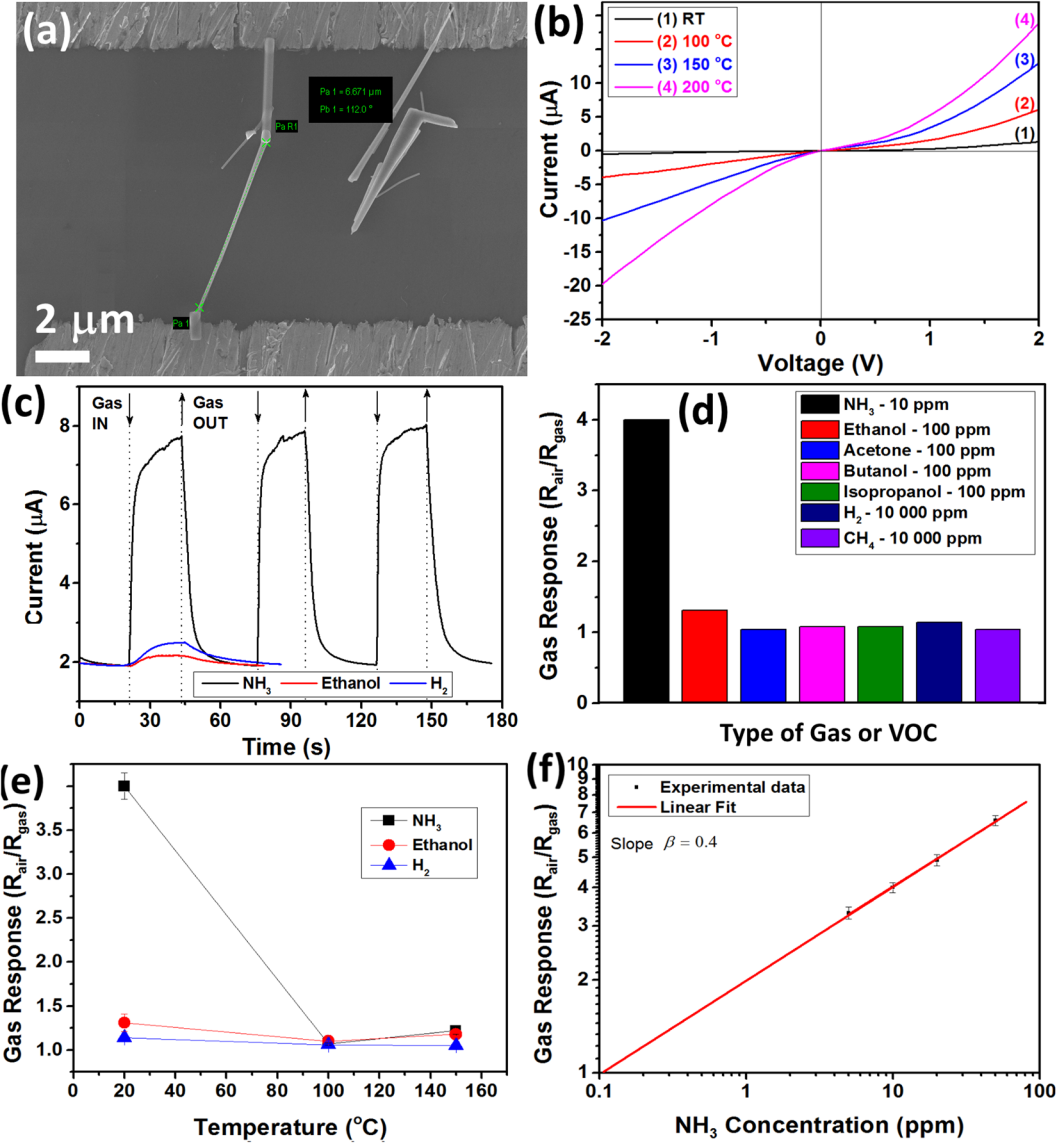
(**a**) SEM image of the nanosensor based on individual ZnO-CNT NW with D = 100 nm (from sample with 2.0 wt% CNT). (**b**) Current – Voltage characteristics of the nanosensor at different operating temperatures. (**c**) Dynamic gas response of the nanosensor at room temperature and applied bias voltage of 2 V to 10 ppm of NH_3_, 100 ppm of ethanol and 10 000 ppm of H_2_ gas. (**d**) Gas response to different gases and VOCs at room temperature. (**e**) Gas response to 10 ppm of NH_3_, 100 ppm of ethanol and 10 000 ppm of H_2_ gas versus operating temperature. (**f**) Gas response of nanosensor at room temperature versus concentration of NH_3_.

Figure 5a show a SEM image of a nanosensor fabricated using an individual ZnO-CNT NW from samples with 2.0 wt% CNT. The diameter (D) of the NW is about 100 nm, while the length is ~6.6 µm. The NW was contacted to pre-patterned Au/Cr contacts by Pt complex, resulting in the formation of double Schottky contacts (see Fig. 5b). A SEM image with higher magnification of an integrated individual ZnO-CNT NW is presented in Supplementary Fig. S6a, showing the presence of CNTs on the surface of ZnO NW. The *I*-*V* characteristics at different temperatures of the nanosensor are presented in Fig. 5b, showing a semiconducting behaviour. The device was tested to different gases and VOCs at different operating temperatures. Figure 5c shows the dynamic response at room temperature to 10 ppm of NH_3_, 100 ppm of ethanol vapours and 10 000 ppm of H_2_ gas. The nanosensor showed excellent repeatability and complete recovery to the initial electrical baseline. The applied bias voltage was 2 V. The room temperature gas response to other VOCs is presented in Supplementary Fig. S6b. The calculated gas response of our nanosensor is presented in Fig. 5d, showing the excellent selectivity to NH_3_, as in the case of ZnO-CNT networks (see Fig. 3b). The gas response to 10 ppm of NH_3_, 100 ppm of ethanol, acetone, butanol and isopropanol vapours and 10 000 ppm of H_2_ and CH_4_ gas is ~4, 1.31, 1.04, 1.08, 1.08, 1.14 and 1.04, respectively. The calculated response time and recovery time for NH_3_ response is beyond 10 s and 6 s, which is faster compared to the data obtained for ZnO-CNT networks and can be explained based on higher accesibility of the individual NW surface to the gas analyte without neccesity of gas diffusion through the networks. From Fig. 5f the LDL of the nanosensor was calculated, showing a value below 0.2 ppm or 200 ppb, which is slightly lower than in the case of the inverstigated 3D networks. The calculated gas response of nanosensors based on NW with D ~100 nm to 5, 10, 20 and 50 ppm of NH_3_ is 3.3, 4, 4.9 and 6.6, respectively (see Fig. 5f). The slope *β* = 0.4.

**Figure 6:**
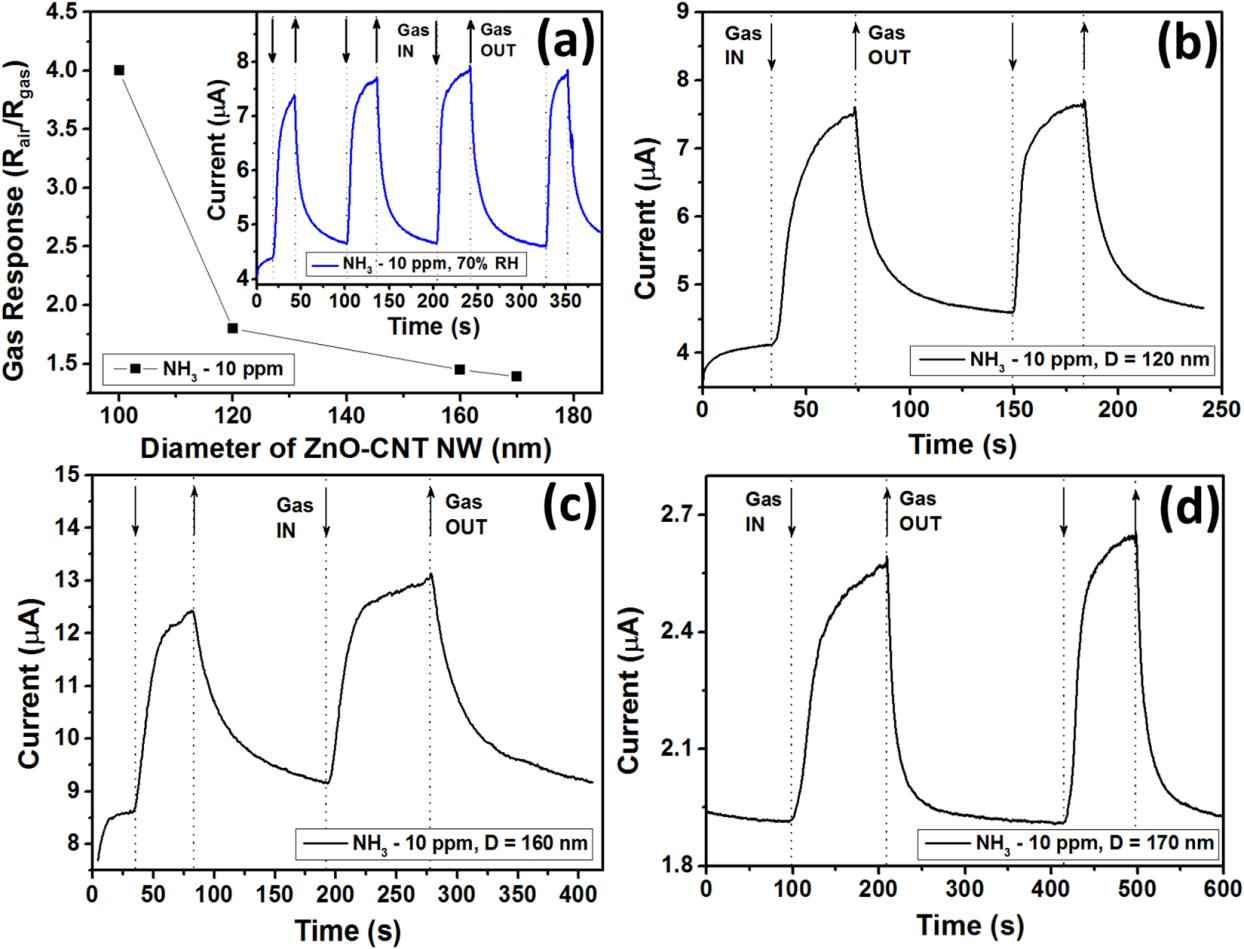
(**a**) Gas response at room temperature to 10 ppm of NH_3_ of nanosensors based on individual ZnO-CNT NWs (from samples with 2.0 wt% CNT) versus diameter of NW. In the inset the room temperature dynamic NH_3_ response (10 ppm) of nanosensor based on NW with D = 100 nm under 70% RH is presented. Dynamic gas response to 10 ppm of NH_3_ at room temperature of nanosensor based on NW with: (**b**) D = 120 nm; (**c**) 160 nm; and (**d**) 170 nm.

The influence of the diameter of individual ZnO-CNT NWs on gas sensing properties was also investigated. Thus, other ZnO-CNT NWs with D ~120, 160 and 170 nm were integrated in nanosensor devices using the same procedure and from the same samples (ZnO-CNT networks with 2.0 wt% CNT). The SEM images of respective nanosesnors are presented in Supplementary Fig. S7. The gas response at room temperature to 10 ppm of NH_3_ versus the diameter of the NWs is presented in Fig. 6a (the applied bias voltage was 2 V). The decrease in gas response from 4 to 1.8, 1.45 and 1.39 was observed by increasing the diameter of NW from 100 nm to 120, 160 and 170 nm, respectively. The dynamic response of nanosensors to 10 ppm of NH_3_ at room temperature for 120, 160 and 170 nm is presented in Fig. 6b–d, respectively. The calculated response and recovery time for NW with D = 120 nm is ~17 s and 30 s, for D = 160 nm is 31 s and 50 s, and for D = 170 nm is 52 s and 54 s, respectively, confirming the evidence of slowing the rapidity by decreasing the diameter of the hybrid nanowire (see Supplementary Fig. S8).

## Discussion

The decrease in gas response by increasing the diameter of the nanowires can be directly related to lowering the influence of transport properties of the nanowire by surface phenomena. Many studies demonstrated the importance to use thinner nanostructures for fabrication of nanosensors using different types of metal oxides. For example, Lupan *et al*. experimentally showed the increase in the VOCs response of individual MoO_3_ nanobelts by decreasing in the NW diameter down to 150 nm^18^. Another interesting example was demonstrated with nanosensors based on individual Fe_2_O_3_ NWs^48^. Authors integrated the Fe_2_O_3_ NWs with diameter down to ~25 nm into nanosensors device using FIB/SEM and showing the exceptional improvements in acetone vapour sensing at room temperature by decreasing the NW diameter. Also, the integration of CuO NW with diameter of ~50 nm into nanosensor device showed ultra-fast and highly-sensitive detection of ethanol vapours at room temperature^26^. However, more results are reported based on ZnO NWs^17,44,49^.

**Figure 7:**
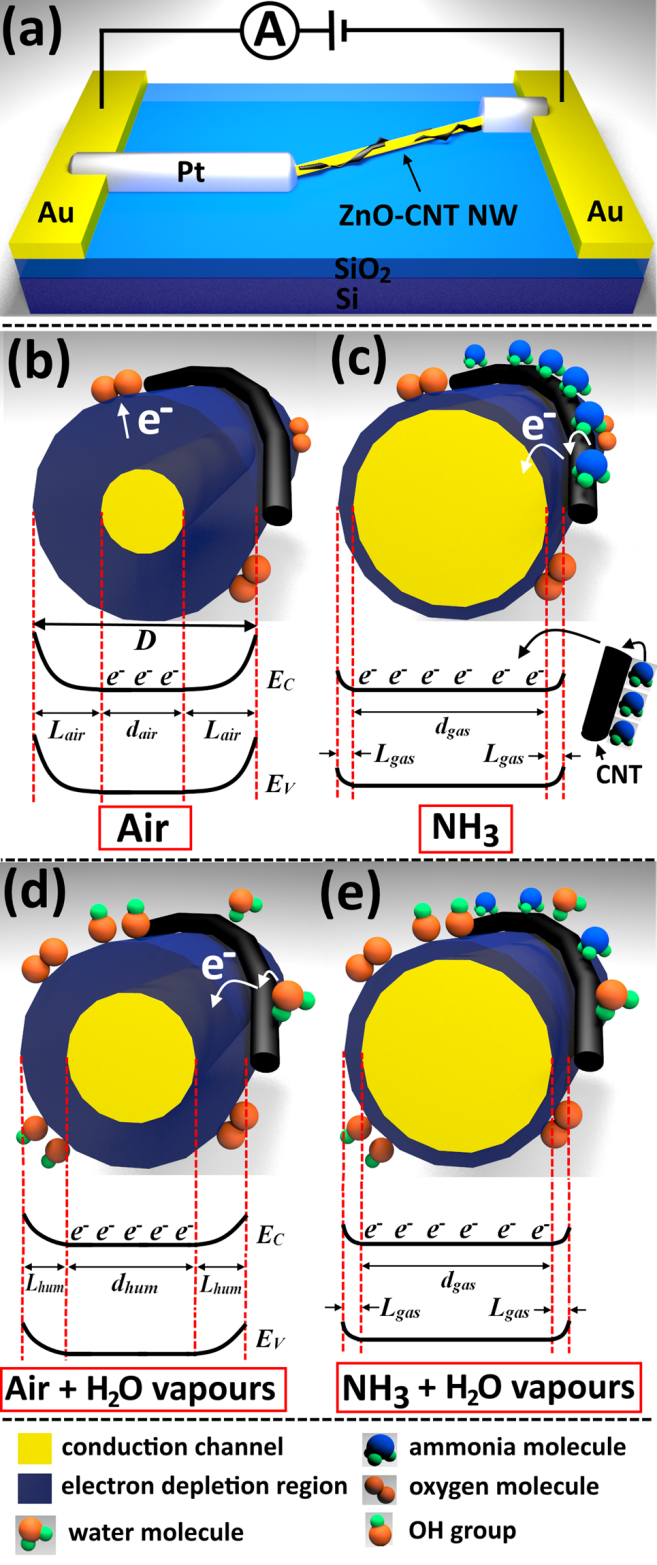
(**a**) Schematic illustration of the nanosensor structure’s based on individual ZnO-CNT NW and the chosen connection for electrical measurements. (**b**–**e**) Proposed NH_3_ sensing mechanism with energy band diagrams at room temperature of individual ZnO-CNT NW without taking into account the oxidation of NH_3_ molecules by oxygen species. (**b**) Exposure under ambinet air by ionization of oxygen molecules at surface of the NW and formation of the electron depletion region with width *L*_*air*_. The width of conduction channel was noted as *d*_*air*_. (**c**) Exposure under ammonia by adsorption of NH_3_ molecules on the surface of CNT and subsequent electron transfer to ZnO NW, leading to a narrowing of the electron depletion region (*L*_*gas*_) and widenning of the conduction channel (*d*_*gas*_). (**d**) Exposure under air with high concentration of water vapours. Water molecules adsorb on the surface of ZnO forming OH groups and on the surface of CNTs. Both reactions have donor effect, leading to a narrowing of the electron depletion region (*L*_*hum*_) and widenning of the conduction channel (*d*_*hum*_). (**e**) Exposure under NH_3_ molecules and high concentration of water vapours, leading to practically the same results as in the case of (**c**).

Figure 7a shows a schematic illustration of a nanosensor device based on individual ZnO-CNT NW and respective connection for electrical measurements. Under assumption of coaxial geometry of ZnO NW, the conductance under exposure in air (*G*_*air*_) and NH_3_ (*G*_*gas*_) can be expressed by equations (7 and 8)^50^:

7 $${G}_{air}=q{N}_{D}{\mu }_{n}\frac{\pi {(D-2{L}_{air})}^{2}}{4l}$$

8 $${G}_{gas}=q{N}_{D}{\mu }_{n}\frac{\pi {(D-2{L}_{gas})}^{2}}{4l}$$

where *µ*_*n*_ is the electron’s mobility, *l* is the length of NW and *L*_*air*_ and *L*_*gas*_ is the width of electron depleted region under exposure in air and NH_3_, respectively (see Fig. 7b,c). The width of electron depleted region can be expressed generally as (equation (9))^17,50^:

9 $$L={\lambda }_{D}{(\frac{q{V}_{s}}{kT})}^{1/2}$$

where *λ*_*D*_ is Debye length. Thus, it can be concluded that the conductivity of individual NWs is highly dependent on thevariation of the electron depletion region and *V*_*s*_. For *L*_*air*_ being comparable with *D*/2, a higher modulation in conductance of NWs can be obtained, i.e. the surface phenomena have a greater influence on transport properties . This explains the higher sensing performances of individual strucutres with lower diameter.

Varying the *V*_s1_ from 0.1 to 3 V the *L*_air_ for pristine ZnO NW was calculated to be ~20–50 nm^32,44^. Thus, assuming that *L*_gas_ is much lower than *D* of NW, the gas response can be expressed by equation (10)^50^:

10 $$S=\frac{{R}_{air}}{{R}_{gas}}={(\frac{D}{D-2{L}_{air}})}^{2}={(\frac{D}{D-2{\lambda }_{D}{(\frac{q{V}_{s1}}{kT})}^{1/2}})}^{2}$$

Therefore, compared to ZnO-CNT networks the influence of the potential barriers height between NWs is excluded in case of individual NW and the gas sensing mechanism is based mainly on the modulation of the electron depletion region (see Fig. 7b,c). However, the higher sensing properties with respect to NH_3_ can be explained using the same principles as described for the networked ZnO-CNT NWs, i.e. transfer of electrons to ZnO NWs as a result of NH_3_ molecules adsorption on the surface of CNTs (see Fig. 7b,c). Thus, under exposure to air the oxygen species are adsorbed on the surface of the ZnO-CNT NW forming the electron depletion region with width *L*_air_ (see Fig. 7b). Under exposure to NH_3_, the electrons donated from CNTs after adsorption of NH_3_ molecules decrease the width of the electron depletion region, *L*_gas_ (see Fig. 7c). Thus, due to the presence of CNTs, the modulation in *L* is higher, giving rise to higher gas response. In our case such high performances can be explained on the very thin diameter of integrated NWs (down to 100 nm).

The influence of RH on gas response of the nanosensor fabricated using NW with D = 100 nm was also investigated. The inset from Fig. 6a shows the dynamic NH_3_ response to 10 ppm at room temperature and 70% RH. The calculated gas response is 1.78, which is with ~45% lower than gas response at 35% RH. The calculated response and recovery time is 22 s and 42 s, respectively. Thus, the rapidity under 70% RH is greatly lowered. The main reason for the decreaes in rapidity and gas response is the same as in the case of the 3D networks mentioned before, namely the hydroxyl poisoning (Supplementary equation (S1))^6^. Thus, under exposure to ambient air with high RH value, OH groups will form on the surface of ZnO NW, causing the increase in the current due to donated electrons after reaction from Supplementary equation (S1), as well as after adsorption of water molecules on CNTs (see Fig. 7d). As can be observed from Fig. 5c and inset of Fig. 6a, the current under exposure to air increased approximately twice in its magnitude. In this case, the electron depletion region is lower (*L*_*hum*_ < *L*_*air*_), and the conductivity is higher (equations (7) and (8)). Under exposure to ammonia, less molecules will adsorb on the surface of the CNTs, due to already adsorbed water molecules, leading to a lower gas response as a result of a lower modulation of the electron depletion region (see Fig. 7e and equations (7 and 8)).

Supplementary Table S3 shows the recent results on NH_3_ gas sensors based on individual nanostructures of metal oxides and other materials, such as carbon based nanomaterials and polymers, which are known to be good sensors for ammonia and VOCs. As can be observed, our sensor demonstrates much higher gas response compared to the  other presented results, as well as essentially higher rapidity, esspecially for recovery. Thus, the nanosensors based on individual ZnO-CNT NWs are excellent candidates in household and outdoor air quality monitoring applications for fast and realiable detection of ammonia. Also, the low power consumption of nanosensors combined with possibility to work at room temperature, obviously increase their attractiveness compared to conventional gas sensor structures on metal oxides with integrated micro-heaters and relatively much higher power consumption. Overall, our investigations suggest that the addition of CNTs to highly porous ZnO NW networks is a very efficient strategy to essentially improve the sensing properties for efficient detection of ammonia and several other gass which could be helful for developing new generations of devices for climate monitoring and other applications.

## Methods

The ZnO NW networks were synthesized by an ultrafast modified flame transport synthesis, the normal flame transport synthesis is described in our latest work^24,25^ at which Zn powder placed in a crucible was inserted into a preheated to 900 °C muffle furnace with a N_2_ saturated atmosphere. After few minutes needed for melting and vaporizing of Zn precursor (oxidation being prevented by N_2_ atmosphere) the gas flow was changed to pressured air and a rapid oxidation took place. The duration of the whole process is about 10–15 min. After NW production, the obtained loose NW powder is pressed into cylindrical pellets (*h* = 3 mm, *d* = 6 mm) with a density of 0.3 g/cm³ and reheated to 1150 °C for 5 h, thereby sintering the interconnection points of the NWs A detailed view of the networks’ morphology can be seen in Fig. 1. For the CNT infiltration of the highly porous ceramic ZnO networks a commercial available aqueous CNT dispersion (CarboByk 9810) is used, which is diluted with distilled water to contain 0.1 wt% of CNTs. The dispersion is treated by a sonication in an ultrasound bath for 20 min to reduce the amount of agglomerates. After that the dispersion is placed in a computer-controlled syringe and slowly dropped (130 µl) on the ceramic networks. Subsequently the filled ceramic sponges are dried for one hour. To increase the amount of CNTs, the process can be repeated several times^24,25^ (Fabian Schütt, Stefano Signetti, Helge Krüger, Sarah Röder, Daria Smazna, Sören Kaps, Stanislav N. Gorb, Yogendra Kumar Mishra, Nicola M. Pugno, Rainer Adelung Nat. Commun. 2017 in press, doi:10.1038/s41467-017-01324-7). The characterization of pristine ZnO NW networks was reported in previous work^23–25^ (Fabian Schütt, Stefano Signetti, Helge Krüger, Sarah Röder, Daria Smazna, Sören Kaps,Stanislav N. Gorb, Yogendra Kumar Mishra, Nicola M. Pugno, Rainer Adelung Nat. Commun. 2017 in press, doi:10.1038/s41467-017-01324-7). The morphological characterization was carried out using scanning electron microscopy (SEM) REM-ZEISS (at 7 kV), while chemical composition of the ZnO-CNT NW networks was examined by EDX spectroscopy. The sensor devices were fabricated using the procedure described in our previous work^37^. The material was mounted between two pre-patterned gold contacts on glass substrate, followed by connection with silver paste. The gap between contacts was ~100 µm. The nanosensor fabrication procedure was described in detail in previous works by Lupan *et al*.^17,18,20,32^. We integrated several single ZnO NWs covered/hybridized with CNT and with different diameters in FIB/SEM system in order to investigate the influence of diameter on sensing performances. The UV and gas sensing measurements were performed at room temperature as previously reported^1,24^. The intensity of UV light (λ = 365 nm) was set to 10 mW·cm^−2^. To tested gases were mixed with ambient air (relative humidity, RH 35%) in order to create the necessary concentration. The total flux was maintained at 500 sccm. The electrical measurements were performed with computer controlled Keithley 2400 source meter. The gas response was defined as $S={I}_{gas}/{I}_{air}$, while UV response was defined as ${S}_{UV}={I}_{UV}/{I}_{dark}$, where *I*_gas_, *I*_air_, *I*_UV_ and *I*_dark_ is the current under gas, air, UV light and dark exposure, respectively.

## Electronic supplementary material

Supplementary Information
