# Supplementary material for: Sensing performances of pure and hybridized carbon nanotubes-ZnO nanowire networks: A detailed study
Source: Sci Rep. 2017 Nov 7;7:14715. doi: 10.1038/s41598-017-14544-0 (PMC5677033; doi:10.1038/s41598-017-14544-0)
Supplement: Supplementary file 1 — Supplementary Information [file 41598_2017_14544_MOESM1_ESM.pdf]

Supplementary Information for

Sensing performances of pure and hybridized carbon nanotubes-ZnO nanowire networks: A detailed study

Oleg Lupan,[1,2,] Fabian Schütt,[1] Vasile Postica,[2] Daria Smazna,[1]*

Yogendra Kumar Mishra,[1,] & Rainer Adelung,[1,*]*

[1] *Institute for Materials Science, Christian-Albrechts Universität zu Kiel, Kaiser Str. 2, D-24143, Kiel, Germany*
[2] *Department of Microelectronics and Biomedical Engineering, Technical University of Moldova, 168 Stefan cel Mare Av., MD-2004 Chisinau, Republic of Moldova*

KEYWORDS: Hybrid network devices, carbon nanotubes, zinc oxide nanowires, ammonia.

Supplementary Text S1.

The influence of applied bias voltage and RH on UV response

We choose only the sample with 0.4 wt% CNT due to the higher rapidity (and only 5 times lower UV response) compared to those of samples with 2.0 wt% CNTs, which is more important for real applications. **Supplementary Figure S4a** shows the UV dynamic response at different applied bias voltages (1 – 20 V) of devices fabricated using ZnO-CNTs (0.4 wt% C) samples. The calculated UV response is presented in **Supplementary Fig. S4b**, showing an increase in UV response from 270 to 1350 by increasing the applied bias voltage from 1 V to 10 V. Thus, the optimal applied bias voltage for ZnO-CNTs is in the range of 10 – 20 V. The same tendency was observed for other types of devices (not shown here). **Supplementary Figure S4c** presents the normalized UV response at different applied bias voltages in order to analyze the influence on the rapidity of the device. However, no considerable change in rapidity was observed. More interesting results were obtained by studying the influence of RH on the UV sensing properties of the ZnO-CNT networks. **Supplementary Figure S4d** shows the dynamic UV response at different values of RH. The RH was set using a bubbling system as reported previously[1]. The considerable increase in dark current by rise in RH value from 35% to 85% was observed, which can be attributed to release of electrons after dissociative adsorption of H_2O molecules on the surface of ZnO NWs[2]. Thus, the adsorbed oxygen species are substituted by H_2O molecules and the electrons captured by oxygen species are released according to **Supplementary equation (S1)**[2,3]:

$$H_2O + O_O + 2Zn_{Zn} \leftrightarrow 2(OH - Zn) + V_O^{\bullet\bullet} + 2e^- \qquad \text{(S1)}$$

where $V_O^{\bullet\bullet}$ is the vacancy created at an oxygen site, Zn_{Zn} and O_O is zinc and oxygen from ZnO lattice.

On top of the increase in dark current a decrease in photocurrent was also observed (see **Supplementary Fig. S4d**). This can be attributed to the decreased lifetime of photogenerated

electron-hole pairs due to lower band energy bending at the surface of ZnO NWs (because less oxygen species are adsorbed at the surface)[3]. As the results of all these effects, the UV response is considerably decreasing by increasing of RH (see **Supplementary Fig. S4e**). Thus, at 35, 45, 55, 65 and 85% RH the UV response is 1350, 150, 40, 18 and 9, respectively. This is almost a 99% decrease in response by change of RH values with 50%. This is much higher compared to pristine and Sn-doped ZnO nanostructures reported previously[4,5]. In case of pristine ZnO NW networks, ZnO-CNT networks with a CNT content of 2.0 and 4.0 wt% the decrease in UV response is about 65%, 98% and 100% (no response was observed), respectively. Thus, one can conclude that such a decrease in UV response is related to the presence of CNTs, and can be explained based on excellent humidity sensing properties of carbon based materials[6,7]. DFT calculations of Pati *et al.* demonstrated that water molecules adsorption is a physisorption process with the hydrogen of a H_2O molecule forming a weak bond with one of the surface C atoms[7]. CNTs can efficiently adsorb water molecules with donor effect and with a 0.03 e^- charge transfer per a single water molecule[6,7].

Supplementary Figure S4f shows the normalized UV response at different values of RH, showing the considerable increase in rapidity. This can be understood based on essential reduction of oxygen species influence on UV sensing mechanism. It is well known that adsorption/photodesorption of oxygen species is a relatively slow process [3,8]. The detailed mechanism of RH influence on UV sensing properties of ZnO nanostructures was reported in previous works[5,9]. The calculated time constants at different values of RH are summarized in **Supplementary Table S2**. Thus, the time constants decrease considerably from τ_{r1} = 0.73 s, τ_{r2} = 1.14 s, τ_{d1} = 0.18 s, τ_{d2} = 1.1 s at 35% RH to τ_{r1} = 0.22 s, τ_{r2} = 0.22 s, τ_{d1} = 0.03 s, τ_{d2} = 0.77 s at 85% RH.

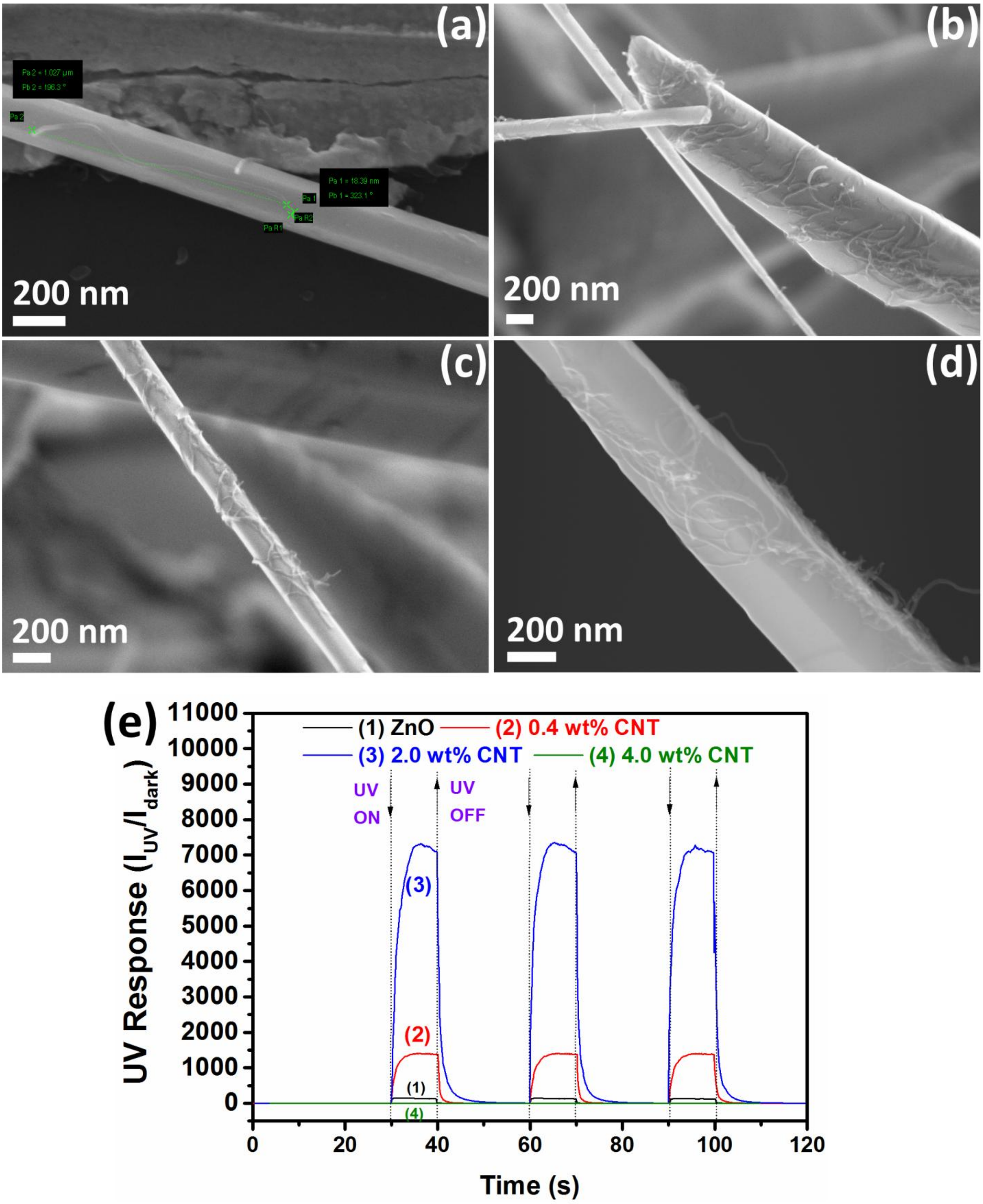

Supplementary Figure S1. (a) SEM image with high magnification of ZnO-CNT NW surface in order to demonstrate the diameter and length of CNTs. (b-d) SEM images of ZnO-CNT NWs surface (2.0 wt% CNT). (e) Dynamic UV response of ZnO-CNT networks with different content of CNT (C in wt%) at 10 V applied bias voltage.

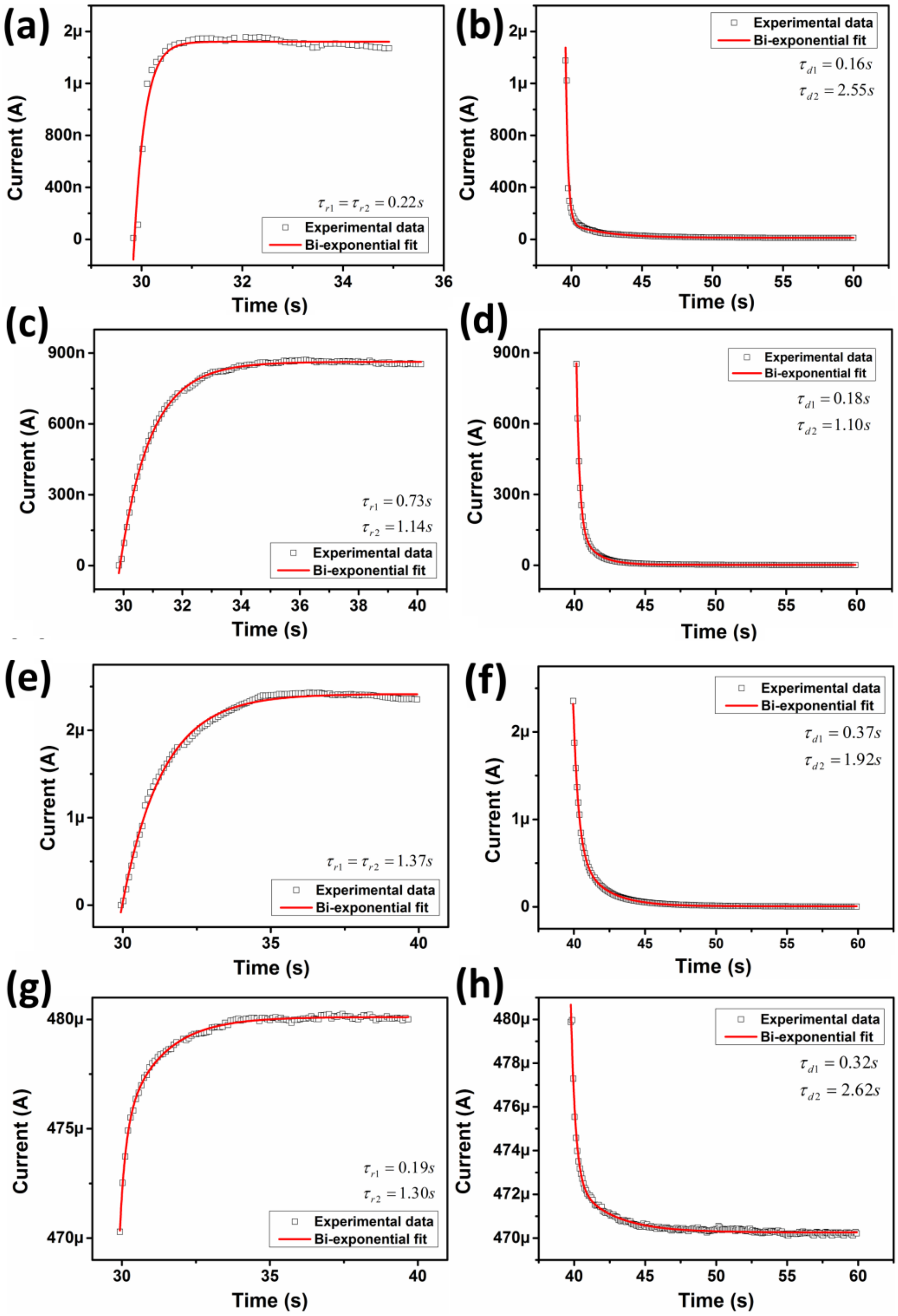

Supplementary Figure S2. Bi-exponential fitting of photoresponse rise and decay curves in order to calculate the time constants for: (a,b) 0 wt% CNTs; (c,d) 0.4 wt% CNTs; (e,f) 2.0 wt% CNTs; and (g,h) 4.0 wt% CNTs, respectively.

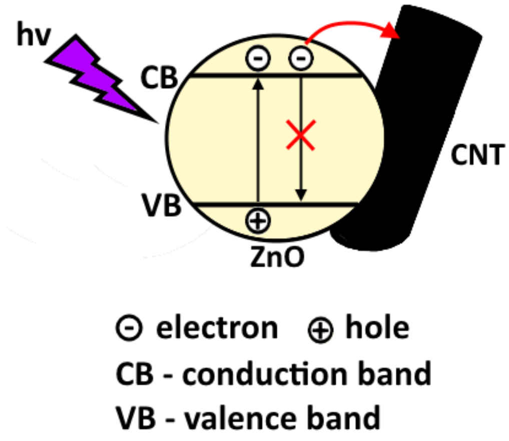

Supplementary Figure S3. Proposed mechanism for photogenerated charge carriers' separation.

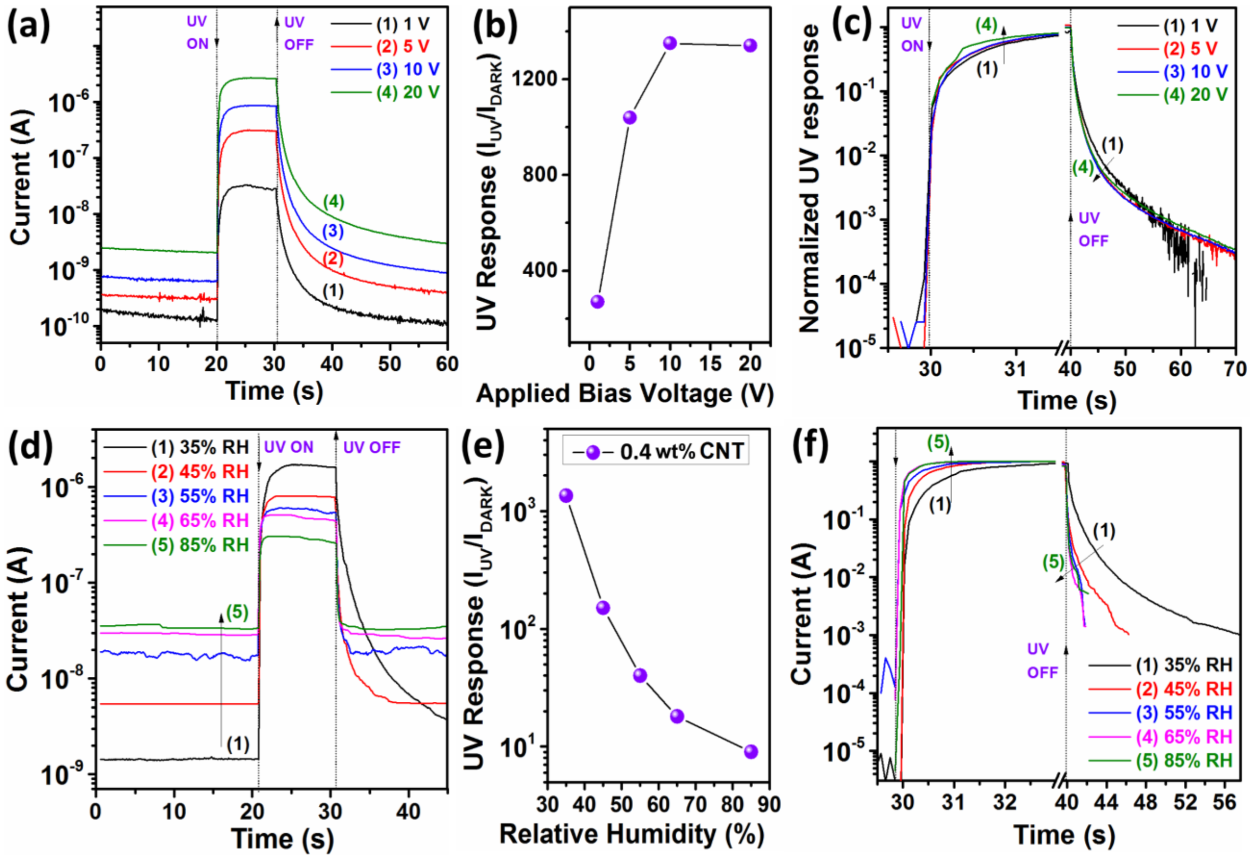

Supplementary Figure S4. (a) Dynamic UV response under the different RH values of device based on ZnO-CNT networks with 0.4 wt% CNT. (b) UV response versus RH value. (c) Normalized dynamic UV response under different RH values. (d) Dynamic UV response under different applied bias voltages of device based on ZnO-CNT networks with 0.4 wt% CNT. (e) UV response versus applied bias voltage. (f) Normalized dynamic UV response under different applied bias voltages.

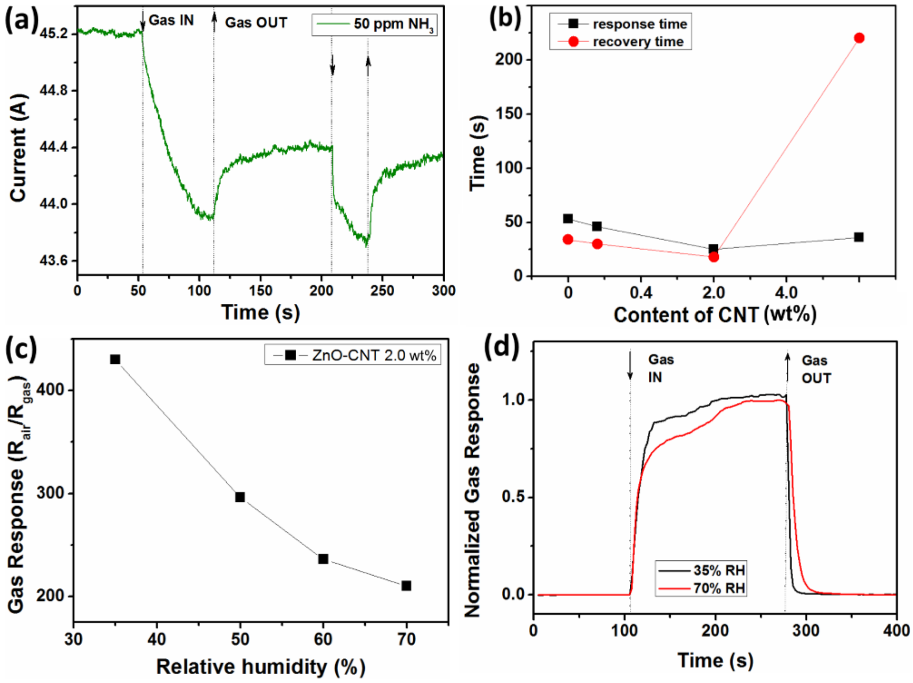

Supplementary Figure S5. (a) The room temperature gas response of samples with 4.0 wt% CNT to 50 ppm of NH_3. (b) The calculated response and recovery time versus content of CNTs in ZnO NW networks. (c) Gas response to 50 ppm of NH_3 at room temperature of sample with 2.0 wt% CNT versus relative humidity. (d) Normalized response of samples with 2.0 wt% CNT to 50 ppm of NH_3 at 35 and 70% RH.

Supplementary Figure S5c shows the gas response to 50 ppm of NH_3 at room temperature versus RH, showing the considerable decrease in gas response by rise of RH. Thus, the gas response is decreasing from 430 to 210 by increasing the RH value from 35% to 70%. This is a decrease of about 50% in gas response with a 35% change of RH. The normalized dynamic response of the device at 35% and 70% RH are presented in **Supplementary Fig. S5d**, showing likewise the decrease in rapidity. Thus, the response and recovery times are increasing from 25 s and 18 s to 97 s and 29 s. The decrease in rapidity and gas sensing response can be corelated with hydroxyl poisoning, namely the lowering in adsorption sites for NH_3 molecules due to adsorption of water molecules[10].

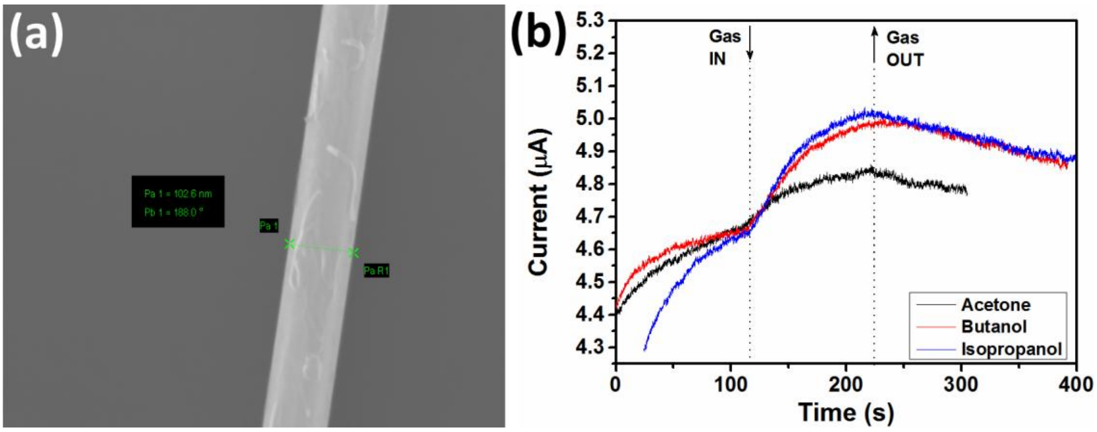

Supplementary Figure S6. (a) SEM image of ZnO-CNT NW surface (D = 100 nm). (b) Gas response at room temperature of nanosensor based on individual ZnO-CNT NW with D = 100 nm (from sample with 2.0 wt% CNT) to 100 ppm of acetone, butanol and isopropanol vapours.

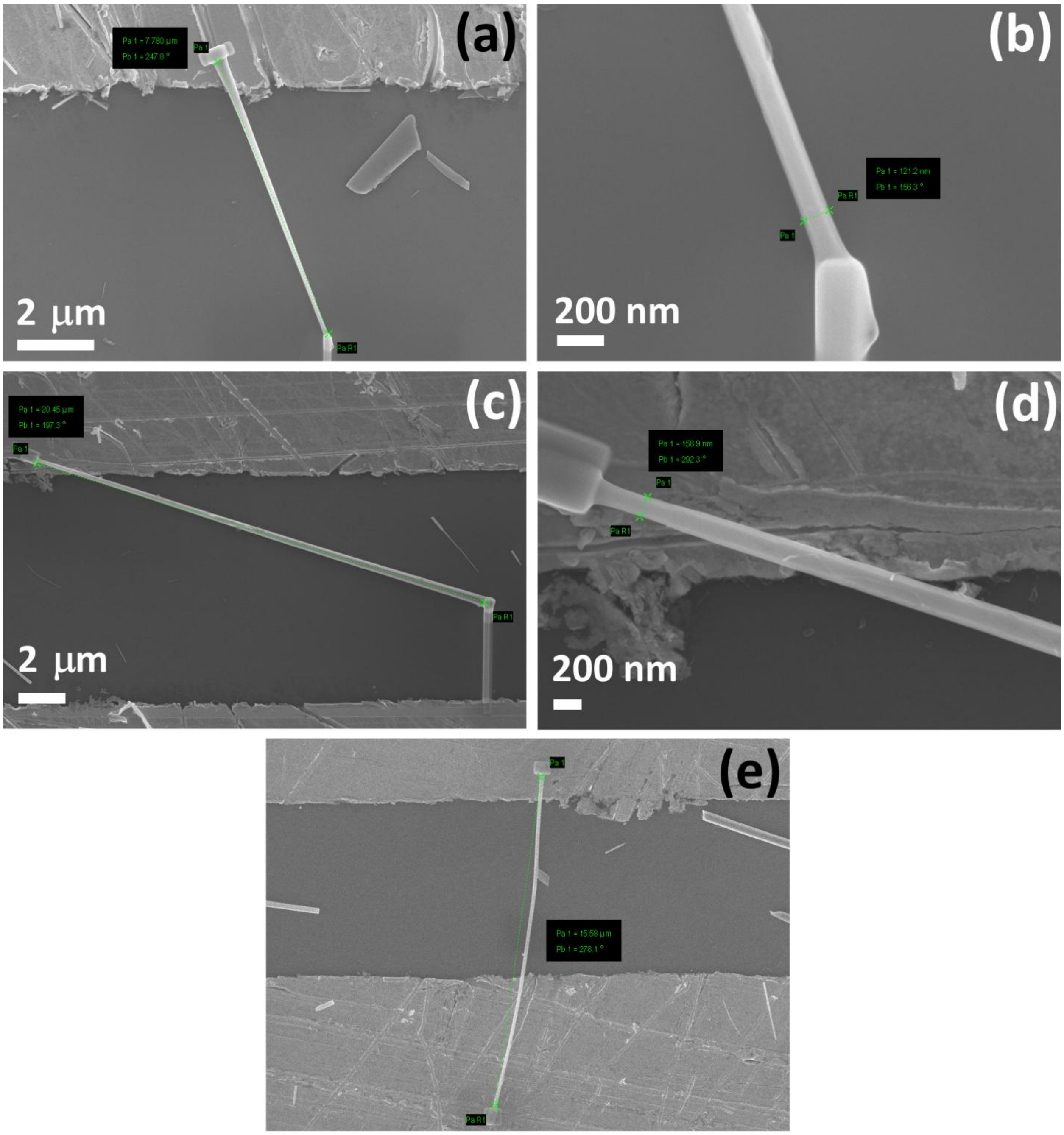

Supplementary Figure S7. SEM images of nanosensors with ZnO-CNT NW with diameter: (a,b) 120 nm; (c,d) 160 nm; and (e) 170 nm.

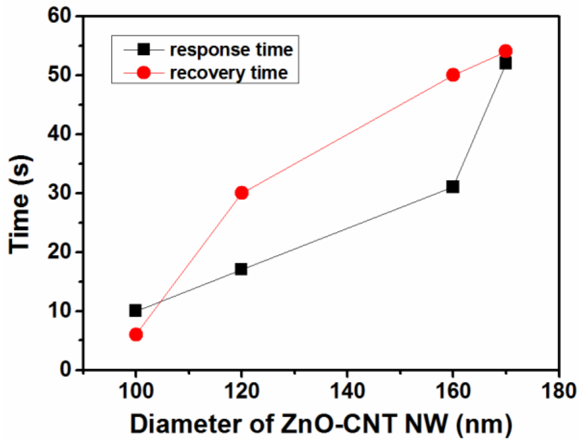

Supplementary Figure S8. Calculated response and recovery time to NH_3 response (10 ppm) for nanosensors based on ZnO-CNT NW with different diameter.

Supplementary Table S1. Calculated values for responsivity (*R*) and internal photoconductive gain (*G*).

**Content of CNT**	**0.0**	**0.4**	**0.2**	**4.0**
**R ($A \cdot W^{-1}$)**	$1.5 \cdot 10^{-5}$	$8.6 \cdot 10^{-5}$	$2.4 \cdot 10^{-4}$	$4.8 \cdot 10^{-4}$
**G**	$5.1 \cdot 10^{-5}$	$2.9 \cdot 10^{-4}$	$8.1 \cdot 10^{-4}$	$1.6 \cdot 10^{-3}$

Supplementary Table S2. Calculated time constants for UV response at different values of RH.

**ZnO-CNT 2.0 wt%**	**Fast rise time constant τ_{r1}, s**	**Slow rise time constant τ_{r2}, s**	**Fast decay time constant τ_{d1}, s**	**Slow decay time constant τ_{d2}, s**
**35% RH**	0.73	1.14	0.18	1.1
**45% RH**	0.55	0.55	0.16	1.02
**55% RH**	0.44	0.44	0.08	0.94
**65% RH**	0.27	0.27	0.04	0.86
**85% RH**	0.22	0.22	0.03	0.77

Supplementary Table S3. NH_3 sensors based on individual structures.

**Type of material and morphology**	**Diameter (nm)**	**NH_3 conc. (ppm)**	**Gas response (R_g/R_a)$^{a)}$ or (R_a/R_g)$^{b)}$**	**Operating temperat. (°C)**	**Response time**	**Recovery time**
Single polypyrrole NW[11]	~ 300	300	~ 1.2 $^{a,c)}$	RT	10 min	-
Single polypyrrole nanoribbon[12]	~ 300	10	~ 1.17 $^{a,c)}$	RT	> 1 min $^{c)}$	> 1 min $^{c)}$
Single dodecyl sulfate doped polypyrrole NW[13]	~ 200	100	~ 1.75 $^{a,c)}$	RT	> 1 min $^{c)}$	> 1 min $^{c)}$
Single polyaniline NW[14]	~ 300	100	~ 55 $^{a,c)}$	RT	~ 15 min	-
Individual SWCNT[15]	~ 1.4	10 000	~ 100 $^{a)}$	RT	~ 1–2 min	-
Individual Ag-functionalized MWCNT[16]	20 - 30	10 000	~ 1.09 $^{a)}$	RT	7 s	> 1 min
Single graphene sheet[17]	-	1800	~ 1.02 $^{a)}$	RT	-	-
Individual Fe_2O_3 NW[18]	~ 25	100	2.86 $^{b)}$	RT	-	-
**Single ZnO-CNT NW**	100	**10**	**4**	**RT**	**10 s**	**6 s**

$^{a)}$ Gas response for *p*-type semiconducting oxides;
$^{b)}$ Gas response for *n*-type semiconducting oxides;
$^{c)}$ Denotes a value approximated from a graphical plot.

Acknowledgments

Dr. Lupan acknowledges the Alexander von Humboldt Foundation for the research fellowship for experienced researchers 3-3MOL/1148833 STP at the Institute for Materials Science, University of Kiel, Germany. The authors acknowledge the support from German Research Foundation (DFG) under SFB 1261 A5. Prof. Adelung and Dr. Lupan gratefully acknowledge partial project funding by the Deutsche Forschungsgemeinschaft contract AD183-17/1, and support from the EU in the framework of the Graphene Flagship. This research was partly supported by the STCU within Project 6229.

References

1 Cretu, V. *et al.* Synthesis, characterization and DFT studies of zinc-doped copper oxide nanocrystals for gas sensing applications. *J. Mater. Chem. A* **4**, 6527-6539, doi:10.1039/C6TA01355D (2016).

2 Tai, W.-P. & Oh, J.-H. Humidity sensing behaviors of nanocrystalline Al-doped ZnO thin films prepared by sol–gel process. *J. Mater. Sci. Mater. Electron.* **13**, 391-394, doi:10.1023/A:1016084309094 (2002).

3 Li, Y., Della Valle, F., Simonnet, M., Yamada, I. & Delaunay, J.-J. Competitive surface effects of oxygen and water on UV photoresponse of ZnO nanowires. *Appl. Phys. Lett.* **94**, 023110, doi:10.1063/1.3073042 (2009).

4 Paulowicz, I. *et al.* Synthesis of three-dimensional self-assembled zinc oxide nano- and microstructure networks for application in room temperature UV and ammonia sensing. *ACS Appl. Mater. Interfaces* **In Progress**, doi:10.1002/pssr.201510414 (2017).

5 Postica, V. *et al.* Morphology dependent UV photoresponse of Sn-doped ZnO nano- and microstructures. *Solid State Sci.* **71**, 75-86, doi: 10.1016/j.solidstatesciences.2017.07.008 (2017).

6 Han, J.-W., Kim, B., Li, J. & Meyyappan, M. Carbon Nanotube Based Humidity Sensor on Cellulose Paper. *J. Phys. Chem. C* **116**, 22094-22097, doi:10.1021/jp3080223 (2012).

7 Pati, R., Zhang, Y., Nayak, S. K. & Ajayan, P. M. Effect of H_2O adsorption on electron transport in a carbon nanotube. *Appl. Phys. Lett.* **81**, 2638-2640, doi:10.1063/1.1510969 (2002).

8 Postica, V. *et al.* Multifunctional device based on ZnO:Fe nanostructured films with enhanced UV and ultra-fast ethanol vapour sensing. *Mater. Sci. Semicon. Proc.* **49**, 20-33, doi:http://dx.doi.org/10.1016/j.mssp.2016.03.024 (2016).

9 Schütt, F. *et al.* Single and Networked ZnO–CNT Hybrid Tetrapods for Selective Room-Temperature High-Performance Ammonia Sensors . *ACS Appl. Mater. Interfaces* **9**, 23107–23118, doi: 10.1021/acsami.7b03702 (2017).

10 Lupan, O. *et al.* Hybridization of zinc oxide tetrapods for selective gas sensing applications. *ACS Appl. Mater. Interfaces* **9**, 4084 - 4099, doi:10.1021/jp5038415 (2017).
11 Hernandez, S. C., Chaudhuri, D., Chen, W., Myung, N. V. & Mulchandani, A. Single Polypyrrole Nanowire Ammonia Gas Sensor. *Electroanal.* **19**, 2125-2130, doi:10.1002/elan.200703933 (2007).
12 Chartuprayoon, N., Hangarter, C. M., Rheem, Y., Jung, H. & Myung, N. V. Wafer-Scale Fabrication of Single Polypyrrole Nanoribbon-Based Ammonia Sensor. *J. Phys. Chem. C* **114**, 11103-11108, doi:10.1021/jp102858w (2010).
13 Hangarter, C. M. *et al.* Maskless electrodeposited contact for conducting polymer nanowires. *Appl. Phys. Lett.* **92**, 073104, doi:10.1063/1.2883923 (2008).
14 Liu, H., Kameoka, J., Czaplewski, D. A. & Craighead, H. G. Polymeric Nanowire Chemical Sensor. *Nano Lett.* **4**, 671-675, doi:10.1021/nl049826f (2004).
15 Kong, J. *et al.* Nanotube Molecular Wires as Chemical Sensors. *Science* **287**, 622 (2000).
16 Cui, S. *et al.* Fast and Selective Room-Temperature Ammonia Sensors Using Silver Nanocrystal-Functionalized Carbon Nanotubes. *ACS Appl. Mater. Interfaces* **4**, 4898-4904, doi:10.1021/am301229w (2012).
17 Dan, Y., Lu, Y., Kybert, N. J., Luo, Z. & Johnson, A. T. C. Intrinsic Response of Graphene Vapor Sensors. *Nano Lett.* **9**, 1472-1475, doi:10.1021/nl8033637 (2009).
18 Lupan, O. *et al.* Localized synthesis of iron oxide nanowires and fabrication of high performance nanosensor based on a single Fe_2O_3 nanowire. *Small* **13**, 1602868, doi:10.1021/jp5038415 (2017).
19 Lupan, O. *et al.* Localized Single and Networked CuO Nanowires for Highly Sensitive p-type Semiconductor Gas Sensor Applications. *Phys. Status Solidi RRL* **10**, 260-266 (2016), doi: 10.1002/pssr.201510414 (2017).
